# Project 7A - 7 facts about allergy and asthma for schools

**DOI:** 10.1186/2045-7022-5-S3-P103

**Published:** 2015-03-30

**Authors:** Jan Richter, Jarmila Richterova, Lenka Kubrichtova, Jaromir Bystron

**Affiliations:** 1 Allergology and Clinical Immunology, Trebic, Czech Republic

## 

Allergic diseases and asthma belong to the most common chronic illnesses in childhood. Modern treatment enables a good control of these diseases, in many cases to such a degree that they do not interfere with any everyday activities. People suffering from allergies and asthma are thus sometimes seen as people with a chronic disease which almost everybody suffers from. However, this results in the neglect of situations when their health condition can deteriorate suddenly, sometimes even threaten their life. These complications can also occur at school where allergic children spend a lot of their time.

There is a high standard of health care for children in the Czech Republic thanks to the system of General Practitioners for children and adolescents. However, there are no school nurses or doctors who could manage serious health problems occurring in schools.

In order to ensure better awareness of teachers, in 2009 we prepared a project "7A - 7 facts about allergy and asthma for schools". The initiator and specialist guarantor is the Czech Initiative for asthma (CIPA). It is intended for teachers in nursery, primary and secondary schools.

Since 2009 CIPA organised 79 courses in which 2287 teachers participated; 19 schools obtained the Certificate of a "School friendly for children suffering from allergy and asthma".

